# Impact of crowding on the diversity of expanding populations

**DOI:** 10.1073/pnas.2208361120

**Published:** 2023-03-07

**Authors:** Carl F. Schreck, Diana Fusco, Yuya Karita, Stephen Martis, Jona Kayser, Marie-Cécilia Duvernoy, Oskar Hallatschek

**Affiliations:** ^a^Department of Physics, University of California, Berkeley, CA 94709; ^b^Department of Integrative Biology, University of California, Berkeley, CA 94709; ^c^Genomic Medicine Center, Children’s Mercy Hospital and Research Institute, Kansas, MO 64108; ^d^Department of Physics, University of Cambridge, Cambridge CB3 0HE, United Kingdom; ^e^Biophysics Graduate Group, University of California, Berkeley, CA 94709; ^f^Computational Oncology, Department of Epidemiology & Biostatistics, Memorial Sloan Kettering Cancer Center, New York, NY 10065; ^g^Max Planck Institute for the Science of Light, Max-Planck-Zentrum für Physik und Medizin, Erlangen 91058, Germany; ^h^Peter Debye Institute for Soft Matter Physics, Leipzig University, Leipzig 04103, Germany

**Keywords:** genetic variation, clone size distribution, biofilms, intratumor heterogeneity

## Abstract

Crowding effects critically impact the self-organization of densely packed cellular assemblies, such as biofilms, solid tumors, and developing tissues. When cells grow and divide, they push each other apart, remodeling the structure and extent of the population’s range. Recent work has shown that crowding has a strong impact on the strength of natural selection. However, the impact of crowding on neutral processes, which controls the fate of new variants as long as they are rare, remains unclear. Here, we quantify the genetic diversity of expanding microbial colonies and uncover signatures of crowding in the site frequency spectrum. By combining Luria–Delbrück fluctuation tests, lineage tracing in a novel microfluidic incubator, cell-based simulations, and theoretical modeling, we find that the majority of mutations arise behind the expanding frontier, giving rise to clones that are mechanically “pushed out” of the growing region by the proliferating cells in front. These excluded-volume interactions result in a clone-size distribution that solely depends on where the mutation first arose relative to the front and is characterized by a simple power law for low-frequency clones. Our model predicts that the distribution depends on a single parameter—the characteristic growth layer thickness—and hence allows estimation of the mutation rate in a variety of crowded cellular populations. Combined with previous studies on high-frequency mutations, our finding provides a unified picture of the genetic diversity in expanding populations over the whole frequency range and suggests a practical method to assess growth dynamics by sequencing populations across spatial scales.

## Significance

Growing cell populations become densely packed as cells proliferate and fill space. Crowding prevents spatial mixing of individuals, significantly altering the evolutionary outcome from established results for well-mixed populations. Despite the fundamental differences between spatial and well-mixed populations, little is known about the impact of crowding on genetic diversity. With microbial colonies on plates, we show that the allele frequency spectrum is characterized by a power law for low frequencies. Using cell-based simulations and microfluidic experiments, we identify the origin of this distribution in the volume-exclusion interactions within the crowded cellular environment, enabling us to extend these findings to a broad range of dense populations. This study highlights the importance of cellular crowding for the emergence of rare genetic variants.

Environmental factors often structure the spatial organization of growing cellular populations, such as microbial biofilms (1), bacteria in confined spaces (2–5), developing embryos and differentiating tissues (6), as well as solid tumors (7–10). Advances in lineage tracing techniques are progressively revealing that in many of these cases, growth is nonuniform across the population, as it strongly depends on the mechanical and biochemical cues experienced by each cell (8, 9, 11–18). Nonuniform growth can favor individuals based on their spatial locations rather than their fitness (10, 19–22) and as such can dramatically impact the evolutionary fate of the population.

The interplay between evolution and growth has been extensively investigated in the context of range expansions, in which populations grow by invading surrounding virgin territory (23–29). In cellular range expansions, growth is often limited to a thin layer of cells at the expanding front of the population (the growth layer) due to processes like nutrient depletion, waste accumulation, mechanical pressure, or quorum sensing in the bulk (30–36). Recent studies have revealed that this growth constraint generates an excess of high-frequency mutations in microbial colonies (37) and colorectal cancer xenografts (10). Remarkably, the size distribution of these large clones is exclusively determined by the surface growth properties of the population through a phenomenon called allele surfing (23, 38).

The distribution of low-frequency mutations, however, remains an open question. Assuming a mutation rate of 10^−3^ mutation/genome/generation (typical of microbes) and a population size of 10^8^ to 10^9^ cells, a total of 10^5^ to 10^6^ mutations are generated during population growth. Yet, experimentally only approximately 0.001% of these mutations have been captured by population sequencing in the case of bacterial colonies and tumors (37, 39–41). This suggests that low-frequency mutations constitute the majority of genetic diversity in the population, but since their frequency is often below the detection limit of population sequencing, they go unaccounted for. As a single mutant can be sufficient to drive drug resistance (19), its quantification is imperative to better understand the emergence of resistant cells after drug treatment. While several groups have recently revealed the dynamics of small clones by multicolor lineage tracing in solid tumors (9, 10, 16), a quantitative understanding of the dynamics of low-frequency mutations is still lacking. Here, we address this gap by investigating the dynamics of low-frequency mutations utilizing an expanding microbial colony as a model system.

To probe the low-frequency end of the mutational spectrum, we adapt the classic Luria–Delbrück fluctuation test, normally used to infer mutation rates in well-mixed populations (42), to microbial colonies. We find that the vast majority of mutations occurring during growth are present at very low frequencies and characterized by a clone size distribution that decays faster than that observed at high frequency (37). To investigate the origin and statistics of low-frequency clones at single-cell resolution in a well-controlled environment, we designed a microfluidic chemostat (the “population machine”) that mimics the growth at the expanding front of a colony. In combination with a newly engineered color-switching *S. cerevisiae* strain, we track clonal lineages for ten generations. Visualization of the clones shows that small clones stem from mutations that occur behind the population’s front. The mutant cells are then pushed toward the bulk of the population by the proliferating cells in front and eventually fall out of the growth layer and stop dividing, limiting the maximum size a clone can reach.

Cell-based simulations show that mechanical cell–cell forces are sufficient to explain the observed low-frequency spectrum and that the spectrum’s behavior is robust to cell-level details such as cell shape and mode of division.

We further develop a theoretical model that captures the essential population genetic process that shapes the low-frequency spectrum, extends our results to a broad range of cellular populations, and provides predictions beyond evolutionary neutral populations.

Finally, we discuss a useful sampling strategy to sequence spatially structured populations such as tumors. We show that the spatial position where one takes samples defines which regime of the site frequency spectrum one can capture. Our results suggest that the whole site frequency spectrum can be reconstructed by combining various sampling methods and rescaling.

## Results

### Fluctuation Test in Bacterial Colonies.

**Fig. 1.**
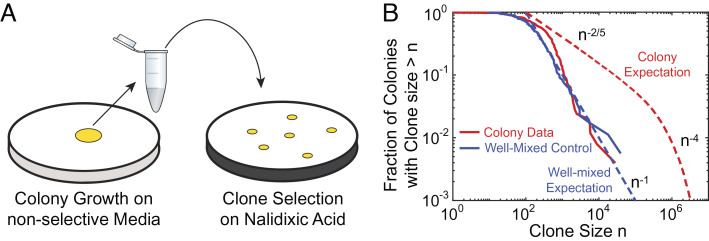
Fluctuation test in bacterial colonies reveals a distinct clone size distribution at low frequencies. (*A*) Fluctuation test on 234 *E. coli* colonies that were grown for 2 d, completely harvested, and then plated on nalidixic acid. The size of clones corresponding to resistant mutations was determined by counting the number of CFUs on selective plates. (*B*) Fraction of the sampled colonies carrying at least *n* resistant mutants (red solid line) in comparison with the well-mixed control (blue solid lines). The blue dashed line corresponds to the classic Luria–Delbrück distribution for well-mixed populations (*n*^−1^) (48), while the red dashed line corresponds to large clones found in colonies *n*^−2/5^ and *n*^−4^ regimes, corresponding to so-called “bubble” and “sector” patterns that were previously characterized (37).

To assess the clone size distribution of small clones (< 10^4^ cells) in *E. coli* colonies grown from single cells to ≈10^9^ cells, we adapted the Luria–Delbrück fluctuation test (42), routinely used to determine spontaneous rates of resistant mutations in well-mixed populations (43–47), to structured populations like colonies (Fig. 1). Colonies were grown on rich nonselective media, scooped up completely after two days of growth, resuspended, and then plated on selective plates containing nalidixic acid (*Methods*). After overnight growth, the selective plates were imaged, and the number of resistant colony-forming units (CFUs) was counted (*Methods*).

The resulting distribution exhibits a decay that resembles the classic Luria–Delbrück distribution typical of well-mixed populations (dashed blue line in Fig. 1), in contrast to the distribution of large mutant clones (> 10^5^ cells) previously observed in similar colonies of the same strain via population sequencing (dashed red line) (37). Indeed, a comparison of the clone size distribution prefactors between colonies and well-mixed populations from sequencing data had previously hinted at the necessary presence of a different distribution regime at very low frequencies (37). In the following, we investigate the physical origin of these low-frequency clones and characterize their statistics.

### Clone Tracking Experiments on Microfluidics.

**Fig. 2.**
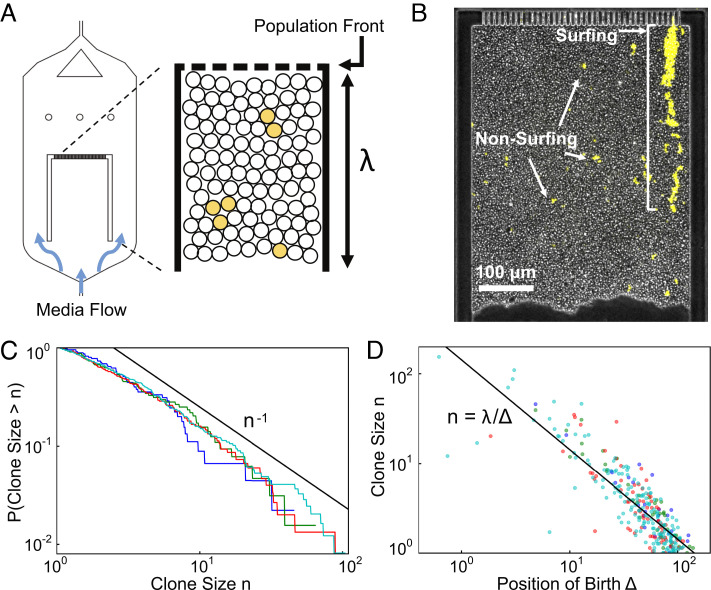
Our microfluidic incubator enables the tracking of front dynamics over several generations. (*A* and *B*) Schematic and snapshot of microfluidic experiments. Cellular growth within the chamber models the comoving frame of the growth layer in an expanding colony. Nutrients are supplied from both the top and bottom of the chamber by diffusion so that all cells grow at a uniform rate (*SI Appendix*, Fig. S4). Cells out of the growth layer are flushed away by continuous media flow. (*C*) Proportion of color-switched cells whose final clone size is greater than *n*, where the area is used as a proxy for clone size. The different lines indicate experimental replicas with, respectively, 45 (blue), 64 (green), 150 (red), and 245 (cyan) mutant clones. (*D*) Relationship between final clone size and distance from the front at which such clone arose. The colors are as in panel (*C*). The black line corresponds to *λ*/*Δ*, where *λ* is the size of the chamber and *Δ* is the distance from the front.

Because in colonies, cell replication is primarily limited to the region near the expanding front, called the “growth layer” (23, 49), most genetic mutations likely occur in this region. In order to visualize the emergence and dynamics of clones over several generations in a well-controlled environment, we designed an in vitro growth layer using a microfluidic chamber inoculated with a newly engineered color-switching budding yeast strain (Fig. 2 *A* and *B* and *Methods*). In the chamber, whose design is inspired by previous studies (50–54), all cells grow at the same rate (*SI Appendix*, Fig. S4) and are continuously pushed out as the cells in front proliferate, mimicking the mechanical interactions between cells at the growing edge of a colony in its comoving frame. By pinning the position of the population front, the device enables tracking the growth layer at single-cell resolution for up to 4 d (Fig. 2 *A* and *B* and *Methods*).

To quantify the dynamics of clones stemming from a single mutational event, we conducted lineage tracking experiments (*Methods* and *SI Appendix*, Fig. S3). Since the switch can occur only at cell division, is inheritable, and does not measurably change the growth rate (*SI Appendix*, Fig. S5), it effectively behaves like a neutral mutation, whose position and growth can be visually tracked with fluorescent microscopy.

During the course of the experiment, we observed both surfing clones, which are born at the very front, as well as nonsurfing clones, which are born behind the front (Fig. 2*B*). Surfing events, which have been previously investigated (37), occur rarely and generate very large clones (10^2^ to 10^3^ cells each) by letting clones stay at the front for some time. By contrast, nonsurfing clones cannot reach sizes larger than 100 cells and exhibit completely distinct dynamics. Using clone area as a proxy for size, we obtained the clone size distribution by tracking nonsurfing clones for 19–50 h. The resulting distribution (Fig. 2*C*) exhibits a power law decay in agreement with the fluctuation test experiments (Fig. 1*B*).

The time resolution of this experiment enables us to go beyond the clones’ ensemble behavior and to track the dynamics of the individual clones. Remarkably, we find that clone size is inversely proportional to the birth position of the first mutant (Fig. 2*D*). This straightforward relationship, despite the complexities of real cellular populations such as cell death, aging of mothers, and feedback of mechanical pressures on growth rate, suggests that a simple physical process may underlie low-frequency clones.

### Mechanical Simulations.

**Fig. 3.**
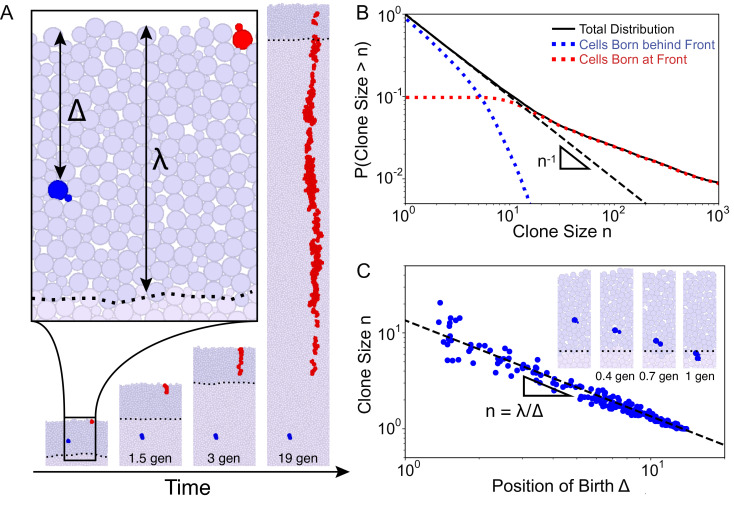
Cell-based simulations show different behaviors between surfing and nonsurfing clones. (*A*) Illustration of the mechanical simulations. Cells lying in the growth layer, defined as the region within a distance *Δ* <  *λ* from the front (the dark purple region with the dashed line showing the back of the growth layer), replicate exponentially. In this image, *λ* = 14 cell widths (about 50 µm). As growth proceeds, the front moves at a constant speed, and cells behind the front are continuously pushed out of the growth layer by replicating cells in front due to excluded-volume interactions. Mutations can either occur at the very front (red cells) generating a surfing clone or behind the front (blue cells) generating nonsurfing clones that are quickly washed out of the growth layer. Clonal dynamics are shown for the first 20 generations of cellular growth. (*B*) The full clone distribution (solid black line) can be subdivided into the size distribution of surfing clones (red dotted line), which dominate the high-frequency tail of the distribution, and nonsurfing clones (blue dotted line), which dominate the low-frequency behavior. The dashed black line shows the *n*^−1^ prediction. (*C*) Scatter-plot identifying for each clone (blue dot) the distance from the front at which the mutation first arose and the final clone size upon exiting the growth layer. Surfing clones are by definition clones that arose within 1-cell distance from the front. Nonsurfing clones are found to satisfy the relationship *n* = *λ*/*Δ*, rationalized in Eq. **1** (dashed black line). The inset shows the dynamics of the blue clone a short time (< 1 generation) after birth in the reference frame of the front. This clone is born at distance *Δ* = 7 cells from the front and grows to a size of *n* = 2.

To gain an intuition into whether the physical growth process alone is sufficient to generate the clone size behavior observed in Fig. 2, we employed 2D mechanical simulations where individually modeled cells proliferate and repel each other upon contact (*Methods*)(35, 55). We introduced an explicit growth layer of finite depth *λ* within which cells of width *σ* grow exponentially at a uniform rate (Fig. 3*A*). Beyond the growth layer, cells are considered to be in the bulk and stop growing. We represented proliferation via budding to mimic our microfluidic budding yeast experiments (Fig. 2).

The clone size distribution obtained from simulations exhibits two regimes (Fig. 3*B*): Very small clones (*n* ≲ *λ*/*σ* in *SI Appendix*, Fig. S1) follow *n*^−1^ while larger clones follow a shallower power law in quantitative agreement with the allele surfing prediction (37). Small clones correspond to mutations originating behind the front whereas large clones correspond to mutations originating at the front. When looking at clones arising behind the front, we find that clone size decreases monotonically with the birth position of the first mutant (Fig. 3*C*).

These results (Fig. 3 *B* and *C*) agree quantitatively with microfluidic experiments (Fig. 2 *C* and *D*), showing that the physical process of population expansion is indeed sufficient to generate the *n*^−1^ low-frequency clone distribution. To further investigate whether clone sizes are dependent on cell-level details, we altered the rules of bud site selection in budding cells and also performed simulations of elongated cells (*SI Appendix*, Fig. S7). In both cases, low-frequency clones decay as *n*^−1^, suggesting that these underlying phenomena may be described by a simple continuum mathematical model.

### Crowding Model of Nonsurfing Clones.

To uncover the physical mechanisms underlying nonsurfing clones, we developed a mathematical model that describes what we observe in the microfluidic experiments and simulations. As in the simulations, we assumed that the growth rate is uniform within a distance *λ* of the expanding front and zero otherwise. We describe clones in a reference frame that is comoving with the expanding front so that rather than accumulating at the edge of the colony, cells are washed out toward the colony bulk (Fig. 3 *C*, *inset*). A mutant of infinitesimal size *δn*_0_ born at a distance *Δ* from the front will grow until it is pushed out of the growth layer by excluded-volume effects from the cells proliferating in front. This happens when the cells in front of the clone have grown to size *λ* to fill the growth layer. Because growth is constant within the growth layer, the mutant will grow by the same relative amount as the layers of cells in front, reaching a final size $\delta n=\frac{\lambda }{\Delta }\delta n_{0}$. By extending this infinitesimal relation to mutant clones with *n*_0_ = 1 cell at the onset of mutation, we have the prediction (*SI Appendix*, section 1 for finite-size analysis)

[1] $$\begin{array}{c} n\approx \frac{\lambda }{\Delta }, \end{array}$$

in agreement with cell-based mechanical simulations (Fig. 3*B*).

**Eq. 1** translates into a prediction for the clone size distribution when combined with the probability of observing a mutation at distance *Δ*. If we assume that the mutation rate is proportional to the growth rate, the probability that a mutation will occur at *Δ* <  *λ* is *P*(*Δ*)=*λ*^−1^. Then, the probability of observing a clone of size *n* is

[2] $$\begin{array}{c} P\left(n\right)=P\left(\Delta \right)\times |d\Delta /dn|=\lambda^{-1}\times \lambda /n^{2}=n^{-2}, \end{array}$$

corresponding to a cumulative clone size distribution of *P*(Clone size > *n*)=1/*n*.

This prediction rests on the assumptions that clone size (*n*) is infinitesimal compared to the growth layer depth (*λ*/*σ*) and that cellular growth rate is uniform within the growth layer. We show in *SI Appendix*, section 1 that Eq. **1** is robust for finite clones up to *n* = *λ*/*σ*, corresponding to mutants born one cell behind the front, which is verified by both microfluidic experiments (*SI Appendix*, Fig. S6) and cell-based simulations (*SI Appendix*, Fig. S1). Additionally, in *SI Appendix*, section 2, we show that our prediction also holds in the case of nonuniform growth inside the growth layer, which we verify via simulations (*SI Appendix*, Fig. S9).

### Reconstruction of Clone Size Distribution from Subsamples.

**Fig. 4.**
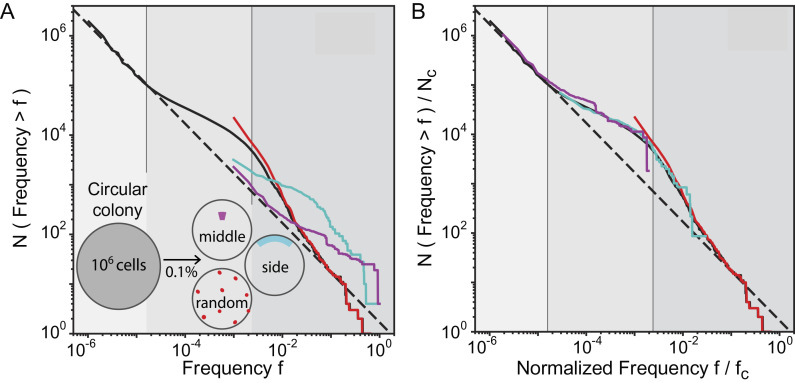
Results from multiple sampling strategies can be combined to infer the mutation rate and growth dynamic of the population. (*A*) Different sampling methods generate distinct clone frequency distributions that highlight distinct properties of the growth dynamics. This is in stark contrast with well-mixed populations where the sampling scheme merely affects how well the clone frequency distribution can be resolved. The solid black line shows the clone frequency distribution (clone size divided by population size) of the whole simulated colony (growth layer *λ*/*σ* = 14 cells) grown up to 10^6^ cells. We identify three frequency *f* ranges in the site frequency spectrum: i) for *f* <  (*λ*/*σ*)/*N*, the distribution is dominated by nonsurfing clones; ii) for (*λ*/*σ*)/*N* <  *f* <  0.003, allele surfing dominates generating bubbles and sectors as previously described; iii) for *f* >  0.003, we see a third behavior, generated by mutations that arise in the first few generations, when the whole microcolony is growing exponentially (*N* <  *π*(*λσ*)^2^). The grayscale regions correspond to nonsurfing bubbles (light gray), surfing bubbles (intermediate gray), and sectors (darkest gray). Sampling 0.1% of the population (equivalent to a 1000X coverage in sequencing) can target nonsurfing small clones and generate their corresponding distribution (middle, magenta) or high-frequency surfing clones (random, red). Sampling an outer segment generates a shifted distribution where distinct trends can be observed. (*B*) These sampling techniques can be combined to reproduce the entire clone size distribution. The rescaling used here requires only knowledge of the total number of cells in the colony and the size/shape of the sampled region, as described in *SI Appendix*, section 8.

By characterizing the behavior of low-frequency mutations, a complete picture of the clone size distribution in crowded expanding populations can now be assessed over the entire frequency range. The full distribution (black line in Fig. 4) exhibits three distinct regimes (gray shades in Fig. 4): two regimes for surfing clones that were previously characterized (37) and one regime for nonsurfing clones at low frequencies characterized in this paper. Using random population sequencing, one can capture the complete distribution only by sequencing unrealistically deeply (over 10^5^X coverage). With a typical coverage (10 to 100X), population sequencing is likely able to assess only the high-frequency regimes (37) (red line in Fig. 4*A*) and misses the nonsurfing bubble behavior that accounts for most of the genetic diversity. However, other sampling strategies can be chosen to take advantage of the spatial proximity of cells that are closely related, a practice that is becoming increasingly frequent in cancer research (40, 41, 56–60). We find that sampling all cells in a small contiguous region of the colony is capable of detecting nonsurfing clones (magenta line in Fig. 4*A*) or the transition between nonsurfing and surfing clones (cyan line in Fig. 4*A*). The data from these contiguous regions can be appropriately rescaled (Fig. 4*B*, *Methods* for rescaling details) in order to recover the complete behavior of the clone size distribution. The local spatial distribution of mutations can therefore be used to identify nonhomogeneous growth in the population by sequencing well-chosen subsamples.

## Discussion

A single resistant cell can seed an entirely new resistant population following an antimicrobial attack. To predict the chances of success of a drug therapy, it is therefore crucial to assess not just the high-frequency mutations but also the rare ones present in small clones after the incubation period. In well-mixed populations, the probability that a mutation carried by at least a frequency *n* is 1/*n* across the entire frequency range. Allele surfing, a hallmark of spatial growth, has been shown to give rise to a different probability distribution characterized by an excess of mutational jackpot events (37). Here, we have shown that, while allele surfing can explain the behavior of large clones, it fails to describe the majority of mutations that reach much lower frequencies.

Crowded growth in dense populations leads to clones whose final size is determined not by when but where a mutation first arose relative to the expanding front. Large surfing clones, which are well described by the surface growth properties of the population, arise at the very front of the expanding edge (49). However, most mutations occur behind the front, are pushed into the population bulk by proliferating cells near the front, and reach only small final clone sizes. This process leads to a reproducible relationship between the final clone size and the initial position of the first mutant cell, generating a clone size distribution different from that of surfing clones.

Because clone size is only determined by the relative position to the front, our argument to derive the full distribution is not limited to two-dimensional colonies expanding at the outer edge but can be applied to a broader class of populations. Theoretical analysis predicts that these results hold in any system where i) growth rate varies only along the direction of expansion, ii) a reference frame exists where the growth profile is constant over time, and iii) the mutation rate per generation is proportional to the growth rate (*SI Appendix*, section 1). Under these conditions, the clone size distribution describing small clones decays like *n*^−1^ up to a critical size that depends only on the growth layer depth but is independent of the number of dimensions (circular colonies vs. solid tumors) or mode of proliferation (budding vs. symmetric division—*SI Appendix*, Fig. S7), demonstrating the robustness of the distribution.

Our theoretical model enables us to make predictions in some evolutionary scenarios that are more complex than that investigated in this paper. For instance, we predict modifications of the *n*^−1^ power law behavior in cases where mutations confer selective effects (*SI Appendix*, sections 3–4 and simulation confirmation in *SI Appendix*, Fig. S8) and where cell death induces a decoupling of mutation and growth rates (*SI Appendix*, section 6).

We also expect deviations from the *n*^−1^ prediction to occur when our model assumptions break down. For instance, our model assumes that the growth rate is time-independent in the frame comoving with the front. This holds for a uniformly growing population, as well as an expanding colony once the growth layer has stabilized (*SI Appendix*, section 2). The time-independence assumption breaks, however, during the transition from uniform growth to a spatially varying but temporally constant growth rate profile. Additionally, our model assumes that cell motion within the growth layer of the colony is purely in the expansion direction, which could break down if long-range collective motion is created via cell motility. Lastly, our model assumes that cell mutation rate is proportional to cell growth rate, which could break down if cells within the colony are stressed and therefore more likely to incur mutations. In such cases, genetic diversity will likely deviate from our predictions, which may be captured by incorporating a spatially varying mutation rate in the model described in *SI Appendix*, section 2.

As the population expands, the majority of mutations are left behind in the bulk, forming a reservoir of genetic diversity in the population. In a typical microbial colony with a growth layer of approximately 100 cells, these mutations would account for more than 99% of the genetic diversity. Analogously, in a solid tumor, they would be responsible for the vast majority of the intratumor heterogeneity, while being largely undetectable by population sequencing. As this class of nonsurfing mutations is the most abundant, it is likely to harbor those rare mutations that can confer resistance. Upon environmental changes that kill the surrounding wild-type cells, for example, due to chemotherapy, these mutants are competitively released (37) and might rescue the population from extinction if cells in the bulk remain viable (61). In the case that cells in the bulk undergo necrosis as can occur in tumors lacking vasculature, we would expect the *n*^−1^ prediction to still hold, but the extent to which these mutants are able to rescue a population would depend on how susceptible cells are to necrosis. High-resolution spatiotemporal studies of rare variants inside tumors would be key to exploring the likelihood of the competitive release scenario.

In well-mixed populations, the detection power is limited by the sequencing coverage one can afford. Still, because the clone size distribution is characterized by a single process across the full range of frequencies, it is possible to estimate mutation rates and selection effects using a reasonable depth of sequencing. Here, we have shown that this procedure cannot be applied to crowded populations growing in space since the shape of the clone size distribution is controlled by very different processes at low and high frequencies. A way around this problem consists in exploiting the spatial arrangement of the population. Neighboring cells are likely to be more closely related than cells farther apart, therefore concentrating sampling power to one or a few locations in the population could reach deeper into the low-frequency regime and measure important population genetic parameters like the mutation rate (*SI Appendix*, section 7) and selection effects (*SI Appendix*, sections 3–4).

In the context of cancer, where there are active debates on how to distinguish selection from neutral evolution (62, 63), our findings highlight the additional challenge of distinguishing selection effects from nonuniform growth that is exclusively driven by spatial constraints. Recent work has recognized similar effects in experiments and simulations, proposing phenomenological models of the tumorogenic evolutionary process (9, 16, 41, 64). Here, we offer a microscopic physical model of evolutionary dynamics, which is consistent with the patterns of genetic diversity in solid tumors *n*^−1^ distribution in (9, 41) and flexible enough to provide insight into the effects that different evolutionary and demographic processes have on the statistics of rare mutants. By taking advantage of the spatial proximity of closely related cells, this model offers rational sampling strategies for probing clone size distributions that can be useful for characterizing intratumor heterogeneity in cancer research (56–60). These results can better characterize the growth dynamics of tumors, which can be used to more precisely identify signatures of selection.

## Materials and Methods

### Fluctuation Test in *E. coli*.

The mutator strain *mutT* of the bacterium *E. coli* was used for the fluctuation test experiment on nalidixic acid. The spontaneous mutation rate in this strain was estimated to be approximately 2 ⋅ 10^−7^ per generation from the fluctuation test in the well-mixed control, which is consistent with previously reported values (65). Colonies starting from single cells were grown on plates with LB and 2% agar at 37 °C for 30 h up to a population size between 10^8^ and 5 ⋅ 10^8^ cells. Each of the 234 colonies was completely scooped from the plate with a pipette tip and resuspended in PBS. A 100X dilution of the resuspension was stored in the fridge for further analysis, while the rest was plated on selective plates containing LB, 2% agar, and 30 µg/mL of nalidixic acid for CFU count. The selective plates were incubated overnight at 37 °C and imaged the following day. The CFU count was determined semimanually with a built-in ImageJ function (below). If selective plates exhibited more than 400 CFUs, the set-aside 100X dilution was itself plated on nalidixic acid, incubated overnight, and imaged the following day to better estimate the size of large mutations. In the control experiment under a well-mixed condition, populations were started from about 50 cells in 200 µL of LB and incubated on a table-top shaker overnight up to saturation. The final population size was estimated to be between 10^8^ and 10^9^. Each of the 178 well-mixed populations was treated similarly as described above.

### Colony Counting on Plates.

Images of colonies on plates were thresholded and binarized using ImageJ. Thresholding was done manually for each image to minimize the effect of noise, such as dust particles, smudges, or glares. Colonies near the rim of the plates were excluded to avoid an edge effect. Colony counting was done automatically with the Analyze particles function of ImageJ. The final clone size of the well-mixed populations control was rescaled by 10 to take into account the different final population sizes and to better visualize the comparison with the data from colonies.

### Mechanical Simulations.

Cells are modeled as 2D rigidly attached disks of width *σ* that proliferate via budding. Upon division, cells divide in polarly, with newly formed buds retaining the orientation of their mothers. Cells interact with each other upon contact via purely repulsive elastic forces and move via overdamped Stokesian dynamics (35). To mimic the diffusion of nutrients into the population from the exterior, we allow only cells within a distance *λ* from the front to actively grow while the rest of the population remains in the stationary phase. In order to simulate a flat geometry, we impose periodic boundary conditions in the horizontal direction so that the population expands outward only in the vertical direction. To calculate the frequency of neutral mutations, we periodically label 40, 000 newly born cells and track their descendants.

### Fabrication of Microfluidics.

The microfluidics was fabricated by soft lithography (66). The master mold was made by spin-coating (CEE 100 spin coater, Brewer Science) a 10-µm-thick layer of negative photoresist (SU8-2010, MicroChem) on a silicon wafer (WaferNet). The photoresist was patterned by photolithography on a mask aligner (Hybralign 200, OAI) through a chrome photomask (Compugraphics). The thickness of the pattern was measured by a stylus meter (Dektak3030, Bruker). Polydimethylsiloxane (PDMS, Sylgard 184, Dow Corning) was mixed with the crosslinker in 10-to-1 ratio and poured on the mold. After being cured at 60 °C overnight, the PDMS was peeled off from the mold and punched holes in for inlets and outlets. The chip was bonded to a glass coverslip after O_2_ plasma treatment by a reactive ion etcher (Plasma Equipment Technical Services). Prior to cell culture, 0.1% bovine serum albumin (Sigma-Aldrich) was loaded into the device to reduce the interaction between cells and the substrate.

### Yeast Strain.

The microfluidics experiments were conducted with the *S. cerevisiae* strain yJK10, derived from strain yDM117 (courtesy of Jasper Rine, University of California, Berkeley). yJK10 employs a Cre-loxP recombination system to switch stochastically from a red (yEmRFP) to a green (yEGFP) fluorescent state, as previously published (37, 67). Using an estradiol-inducible Cre construct allowed us to optimize the average switching rate for our experiments (68). For all experiments, we used a concentration of 1.6 nM *β*-estradiol corresponding to a switching rate of 7.1 ± 4.8 × 10^−4^ per cell per generation (estimated from the number of observed switching during the microfluidics experiments). In principle, the relative fitness between switched and unswitched cells can be set via the differing cycloheximide susceptibility of both states. However, while we did not perform any variation of relative fitness in this study, we chose to use yJK10 to maximize the comparability of our results to ongoing and future investigations involving this strain. Under our experimental condition, the relative fitness between the two states (*s* = 0.022 ± 0.040) is sufficiently small to be neglected (*SI Appendix*, Fig. S5). *SI Appendix*, section 4 for the effect of nonzero *s* on the power law exponent of the distribution of clone size.

### Clone Tracking in Microfluidics.

The microfluidic growth chamber was designed as a population version of the mother machine (54). A suspension of yJK10 cells in an exponential phase was injected into the device with YPD culture medium. After overnight culture, cells grew and filled up the growth chamber. At this point, 1.6 nM *β*-estradiol was added to the culture medium to induce color switching (the switching rate was about 10^−3^ per cell division). Subsequent growth was imaged using time-lapse microscopy on an inverted microscope (IX81, Olympus) with a 10X objective every 10 min for 2 to 4 d. The taken GFP images (color of switched cells) were binarized by Otsu’s method (69), and the dynamics of the clones were manually tracked on Matlab (Mathworks) and ImageJ (NIH). Throughout the experiment, the temperature was controlled at 30 °C by a microscope incubator (H201-T, Okolab), and the flow rate of the medium was regulated by syringe pumps (neMESYS, CETONI) at 15 µL/h. The growth rate of cells was uniform across the chamber under our experimental condition (*SI Appendix*, Fig. S4) (70).

## Supplementary Material

Appendix 01 (PDF)

Movie S1. Experimental 141 tracking of the clone size of the switcher budding yeast yJK10. The growth of switched cells were tracked for ~43 hours. The left panel shows the green fluorescent channel where only switched cells were visible in a jam-packed population (see Fig. 2). The right panel shows the data after the image processing. Clones from a single switching event are labeled by colored circles. The different colors correspond to different switching events.

## Data Availability

Codes in FORTRAN and Matlab, CAD blueprints for microfluidics, and experimental pictures and movies are available on GitHub at https://github.com/Hallatscheklab/Impact-Crowding-Diversity.
